# Astaxanthin Alleviates Early Brain Injury Following Subarachnoid Hemorrhage in Rats: Possible Involvement of Akt/Bad Signaling

**DOI:** 10.3390/md12084291

**Published:** 2014-07-28

**Authors:** Xiang-Sheng Zhang, Xin Zhang, Qi Wu, Wei Li, Qing-Rong Zhang, Chun-Xi Wang, Xiao-Ming Zhou, Hua Li, Ji-Xin Shi, Meng-Liang Zhou

**Affiliations:** Department of Neurosurgery, Jinling Hospital, School of Medicine, Nanjing University, Nanjing 210000, China

**Keywords:** subarachnoid hemorrhage, early brain injury, astaxanthin, apoptosis, Akt/Bad pathway

## Abstract

Apoptosis has been proven to play a crucial role in early brain injury pathogenesis and to represent a target for the treatment of subarachnoid hemorrhage (SAH). Previously, we demonstrated that astaxanthin (ATX) administration markedly reduced neuronal apoptosis in the early period after SAH. However, the underlying molecular mechanisms remain obscure. In the present study, we tried to investigate whether ATX administration is associated with the phosphatidylinositol 3-kinase-Akt (PI3K/Akt) pathway, which can play an important role in the signaling of apoptosis. Our results showed that post-SAH treatment with ATX could cause a significant increase of phosphorylated Akt and Bad levels, along with a significant decrease of cleaved caspase-3 levels in the cortex after SAH. In addition to the reduced neuronal apoptosis, treatment with ATX could also significantly reduce secondary brain injury characterized by neurological dysfunction, cerebral edema and blood-brain barrier disruption. In contrast, the PI3K/Akt inhibitor, LY294002, could partially reverse the neuroprotection of ATX in the early period after SAH by downregulating ATX-induced activation of Akt/Bad and upregulating cleaved caspase-3 levels. These results provided the evidence that ATX could attenuate apoptosis in a rat SAH model, potentially, in part, through modulating the Akt/Bad pathway.

## 1. Introduction

Subarachnoid hemorrhage (SAH) is a medical emergency associated with high mortality and morbidity. Despite major advances in surgical techniques, radiology and anesthesiology, the high rates of mortality and morbidity after SAH have not changed in recent years [1]. Early brain injury (EBI), which refers to the acute injury to the whole brain within the first 72 h after SAH, has been considered as a key factor contributing to overall mortality related to SAH [2]. Thus, the prevention of EBI is considered a major goal in the management of patients with SAH. Although the exact pathogenesis of EBI remains obscure, accumulating studies indicate that apoptosis plays a critical role in the EBI pathogenesis and may represent a novel target for the treatment of SAH [2,3,4]. Increasing evidence has indicated significant levels of apoptotic cell death following SAH, and the beneficial effects of anti-apoptotic therapy in experimental SAH and clinical trials have also been demonstrated [3,4,5]; hence, apoptotic cell death may represent a potential therapeutic target for EBI after SAH.

The serine-threonine kinase, Akt/protein kinase B (PKB), plays an important role in the cell death/survival pathway [6]. It is activated by phosphorylation at the Ser473 residue and acts downstream of the phosphoinositide 3-kinase (PI3K) pathway [7]. Once activated, Akt phosphorylates several target proteins, including the pro-apoptotic protein, Bad, preventing it from binding to Bcl-xL, and inactivating the caspase-3 protein [6,8]. In the central nervous system (CNS), it has been well established that the PI3K/Akt survival pathway is involved in the mechanisms of apoptotic cell death, including cerebral ischemia, traumatic brain injury, spinal cord injury and SAH [9,10,11,12].

Astaxanthin (ATX), a naturally occurring carotenoid, is widely distributed in algae, crustaceans, shellfish and various plants [13]. Recently, accumulating studies have shown that ATX has anti-apoptotic effects in various CNS diseases [14,15,16]. In our previous study, we have found that ATX could reduce apoptotic cell death in an experimental SAH model. However, the molecular mechanisms underlying ATX-dependent inhibition of the apoptotic pathway remain obscure. It has been reported that ATX has the ability to modulate the PI3K/Akt pathway, both* in vivo* and* in vitro* [17,18,19]. Thus, we hypothesized that ATX treatment could modulate the PI3K/Akt survival pathway and alleviate EBI in the early period of SAH.

## 2. Results

### 2.1. General Observation

There were no significant differences in physiological parameters before, during and after surgery. No statistical differences were observed among experimental groups with regard to mean arterial blood pressure, arterial blood gases and body temperature (data not shown).

### 2.2. Mortality, Brain Water Content and BBB Permeability

**Figure 1:**
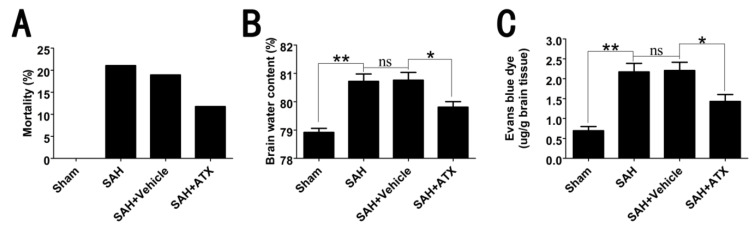
The mortality, brain water content and Evans blue extravasation among each group. (**A**) No rats died in the sham group (zero of 30 rats); eight of 38 rats died in the SAH group, seven of 37 in the SAH + vehicle group and four of 34 in the SAH + ATX group. No significant differences were observed in mortality among each group; (**B**) The brain water content was increased significantly at 24 h after SAH. ATX treatment post-SAH could significantly reduce brain water content when compared with that in the SAH + vehicle group; (**C**) Compared with the sham group, SAH lead to a significant increase in Evans blue extravasation. After ATX administration, the increased blood-brain barrier (BBB) extravasation was markedly reduced as compared with the SAH + vehicle group. Values are expressed as means ± SEM. ** *p* < 0.01, * *p* < 0.05, ^ns^ *p* > 0.05.

The mortality after surgery was 0% (zero of 30) in the sham group, 21.1% (eight of 38) in the SAH group, 18.9 (seven of 37) in the SAH + vehicle group and 11.8% (four of 34) in the SAH + ATX group. There was no significant difference among the SAH, SAH + vehicle and SAH + ATX groups in mortality (Figure 1A).

Brain edema after blood-brain barrier (BBB) disruption is a key event in EBI after SAH. At 24 h, SAH insults could induce a worse brain water content and BBB permeability in comparison with the sham group. There were no significant differences between SAH and SAH + vehicle groups in brain edema and BBB disruption. After ATX administration, the brain edema and BBB disruption were significantly ameliorated as compared with that in the SAH + vehicle group (Figure 1B,C).

### 2.3. Effects of ATX on p-Akt, p-Bad and Caspase-3 Expression

**Figure 2:**
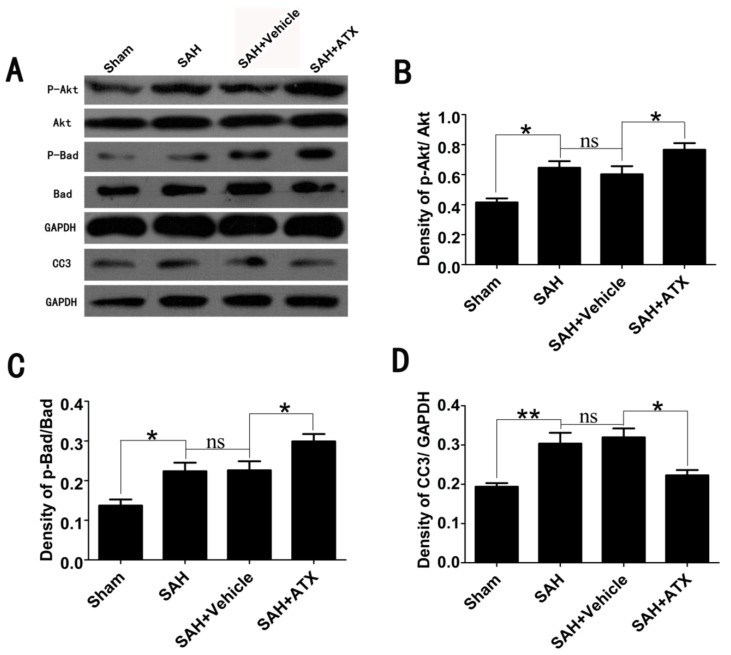
Expression of p-Akt, p-Bad and caspase-3 in the cortex in the sham, SAH, SAH + vehicle and SAH + ATX groups. (**A**) The representative autoradiogram of p-Akt, p-Bad and caspase-3; (**B**–**D**) Quantitative analysis of p-Akt, p-Bad and caspase-3 among all experimental groups. It is shown that SAH could induce a marked increase of p-Akt, p-Bad and caspase-3 expression in the brain samples, as compared with that in the sham group. After ATX administration, the protein levels of p-Akt and p-Bad were further markedly upregulated, whereas protein levels of caspase-3 were significantly downregulated. There was no significant difference between the SAH and SAH + vehicle group in p-Akt, p-Bad and caspase-3 expression. Results are expressed as the means ± SEM. ** *p* < 0.01, * *p* < 0.05, ^ns^ *p* > 0.05.

To determine the influence of ATX on Akt/Bad activation in the cortex after surgery, a western blot analysis was performed. As shown in Figure 2, a similar expression of Akt and Bad was shown among all experimental groups. Densitometric analysis indicated a low level of Akt and Bad phosphorylation in the sham group. The levels of activated Akt and Bad significantly increased in the SAH and SAH + vehicle groups. After ATX administration, the increased p-Akt and p-Bad expression was markedly further elevated as compared with the SAH + vehicle group. There was a low level of caspase-3 expression in the sham group. After SAH insults, the level of caspase-3 was enhanced in the SAH and SAH + vehicle groups when compared with that in the sham group. After ATX treatment, the expression of caspase-3 was markedly reduced as compared with that in the SAH + vehicle group. There were no significant differences in the p-Akt, p-Bad and caspase-3 expression between the SAH group and the SAH + vehicle group.

### 2.4. Effects of ATX on p-Akt, p-Bad and Caspase-3 Distribution

**Figure 3:**
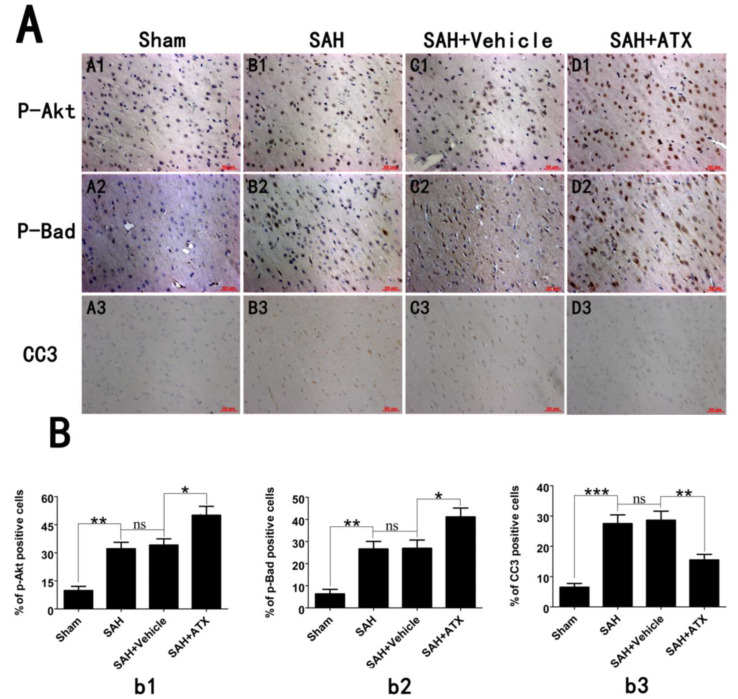
(**A**) Representative photomicrographs showed p-Akt (**A1**–**D1**), p-Bad (**A2**–**D2**) and caspase-3 (**A3**–**D3**) immunohistochemical staining of the temporal lobe in all experimental groups. As shown, low p-Akt, p-Bad and caspase-3 staining in the cerebral cortex was observed in the sham group rats, while strong p-Akt, p-Bad and caspase-3 staining could be seen in the SAH group and SAH + vehicle groups. After ATX administration, the immunoreactivity of p-Akt and p-Bad in the cerebral cortex were further increased following SAH, whereas the immunoreactivity of caspase-3 was significantly downregulated; (**B**) Quantitative analysis of the p-Akt (**b1**), p-Bad (**b2**) and caspase-3 (**b3**) immunohistochemical staining in the temporal lobe in all experimental groups. Values are expressed as the mean ± SEM. *** *p* < 0.001, ** *p* < 0.01, * *p* < 0.05, ^ns^ *p* > 0.05.

The expression and distribution of p-Akt, p-Bad and caspase-3 were identified by immunohistochemistry study. As shown in Figure 3 (A1–D1, A2–D2), positive p-Akt and p-Bad were located mainly at the neurons in the cerebral cortex. The immunoreactivity of p-Akt and p-Bad was weak in the cortex samples in the sham group, with only a few p-Akt- and p-Bad-positive cells in the brain. More p-Akt and p-Bad positively immunostained neurons appeared in the SAH and SAH + vehicle groups. After ATX administration, the number of p-Akt- and p-Bad-positive cells was further increased as compared with the SAH + vehicle group. A few caspase-3-positive cells were observed in the cortex of the sham group, while numerous caspase-3-positive cells stained as brown were evident in the SAH group and SAH + vehicle group. Compared with the SAH + vehicle group, administration of ATX markedly reduced the number of caspase-3-positive cells in the brain at 24 h after SAH (Figure 3A3–D3). These results suggested that ATX treatment could induce phosphorylation of Akt and Bad and suppress caspase-3 expression following experimental SAH.

### 2.5. Effects of ATX on Neural Survival and Cell Apoptosis at 24 h after SAH

**Figure 4:**
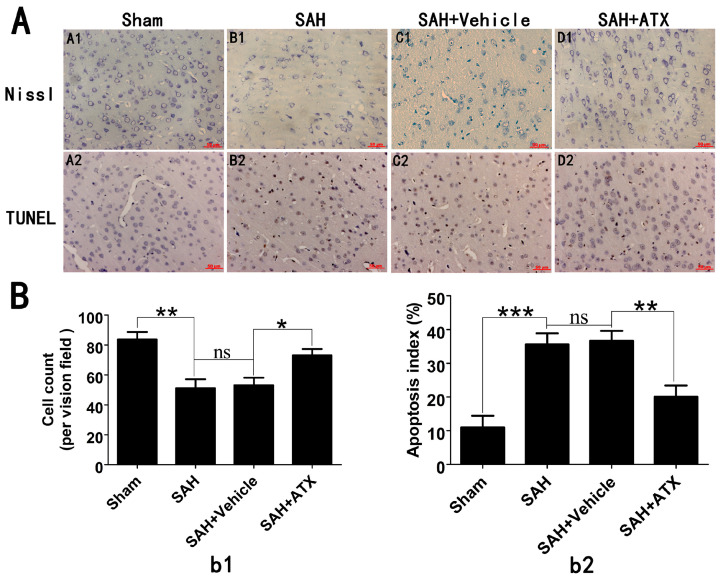
(**A**) Representative photomicrographs of Nissl (**A1**–**D1**) and TUNEL (**A2**–**D2**) staining in the cerebral cortex at 24 h after SAH and (**B**) quantitative analysis of neuronal survival (**b1**) and the apoptotic index (**b2**). As shown in the Nissl staining, in the sham group, the neuronal cell outline was clear and the structure compact, with abundant cytoplasm and Nissl bodies. However, evident neuronal loss and neuronal degeneration were observed in the SAH group and SAH + vehicle groups. Treatment with ATX significantly increased the proportion of surviving neurons. The TUNEL staining showed that the rats in the sham group display rare apoptotic cells in the cortex, while obvious TUNEL-positive cells could be observed in the SAH group and SAH + vehicle groups. In contrast, the proportion of apoptotic cell death decreased significantly in the SAH + ATX group. Values are represented as the mean ± SEM. *** *p* < 0.001, ** *p* < 0.01, * *p* < 0.05, ^ns^ *p* > 0.05.

As shown in Nissl staining (Figure 4A1–D1), evident damage was seen in the SAH and SAH + vehicle groups, with a decrease of cell number, sparse cell arrangements, loss of integrity and dark staining resulting from cytoplasm and karyoplasms. In contrast, after administration of ATX, the severity of neuronal degeneration was markedly alleviated. TUNEL staining (Figure 4A2–D2) showed that rare apoptotic neurons were detected in the sham group. Compared with the sham group, obvious neuronal apoptosis occurred in the SAH and SAH + vehicle groups. After ATX administration, the percentage of TUNEL-positive neurons was significantly decreased following SAH.

### 2.6. LY294002 Abolished the Protective Effects of ATX at 24 h after SAH

**Figure 5:**
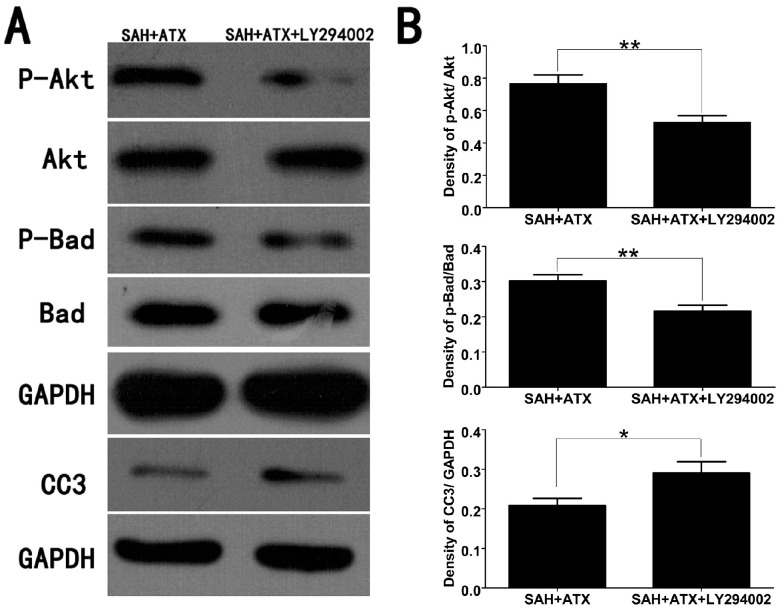
Representative Western blots (**A**) and quantitative analysis of p-Akt, p-Bad and caspase-3 (**B**) in the cortex of the SAH + ATX and SAH + ATX + LY294002 groups. The levels of p-Akt and p-Bad were high in the SAH + ATX group. After LY294002 treatment, the high levels of p-Akt and p-Bad were significantly decreased. In contrast to the low level of caspase-3 in the SAH + ATX group, LY294002 treatment significantly upregulated the level of caspase-3 in the cortex. Results are expressed as the means ± SEM. ** *p* < 0.01, * *p* < 0.05.

**Figure 6:**
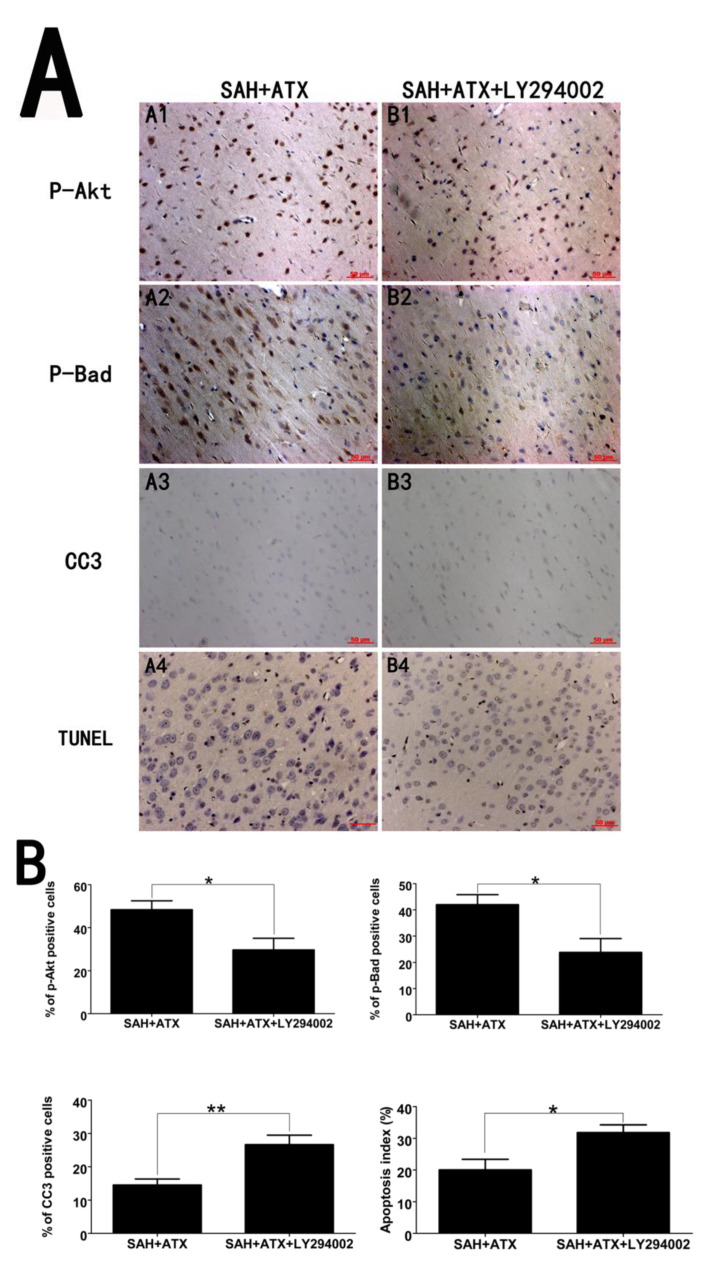
(**A**) Representative immunohistochemical staining for p-Akt, p-Bad and caspase-3 in the cortex of the SAH + ATX and SAH + ATX + LY294002 groups. As shown, there was a strong immunoactivity of p-Akt and p-Bad and slight immunoactivity of caspase-3 in the cortex of the SAH + ATX group. However, after administration of LY294002, the immunoactivity of p-Akt and p-Bad significantly decreased compared with that in the SAH + ATX group. Moreover, LY294002 administration could upregulate the caspase-3 level when compared with that in the SAH + ATX group. These results suggested that the PI3K inhibitor LY294002 treatment could partially abolish the protective effects of ATX in the EBI after SAH; (**B**) Quantitative analysis of the p-Akt, p-Bad and caspase-3 immunohistochemical staining in the cerebral cortex in the SAH + ATX and SAH + ATX + LY294002 groups. Values are expressed as the mean ± SEM. ** *p* < 0.01, * p < 0.05, ^ns^ *p* > 0.05.

LY294002, a specific inhibitor of the PI3K/Akt pathway, was used to further demonstrate the protective effects of ATX after SAH. As shown in Figure 5, the increased expression of p-Akt and p-Bad in the cerebral cortex after ATX administration could be partially abrogated by the PI3K/Akt inhibitor, LY294002. Moreover, LY294002 significantly upregulated the low levels of caspase-3 in the cerebral cortex when compared with that in the SAH + ATX group. Consistent with the western blot results, immunohistochemistry study also showed similar trends of p-Akt, p-Bad and caspase-3 in the experimental groups (Figure 6). As shown, pretreatment with LY294002 could markedly decrease the number of p-Akt- and p-Bad-positive cells and significantly enhance the proportion of positive caspase-3 neuronal cells in the SAH + ATX + LY294002 group. In addition, LY294002 substantially increased the proportion of apoptotic neurons as compared with that in the SAH + ATX group. These results suggested that LY294002 could partially abolish the neuroprotection of ATX by inhibiting the Akt/Bad pathway.

### 2.7. Influence of ATX on Neuronal Survival and Neurological Function within 72 h after SAH

**Figure 7:**
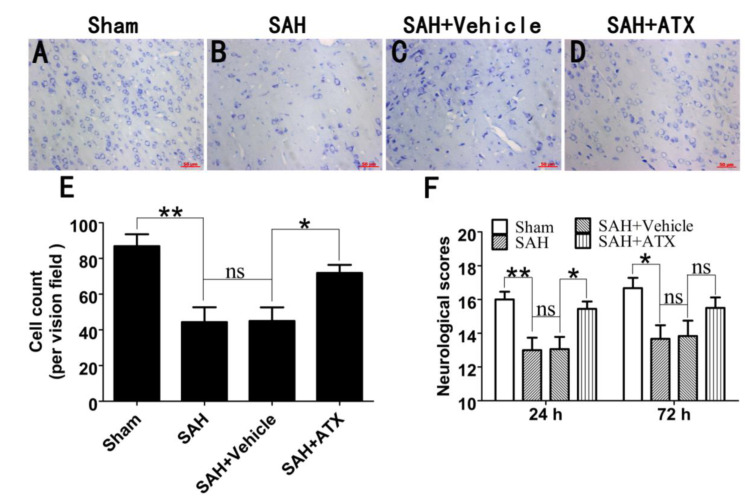
The effect of ATX on neuronal survival and neurological scores within 72 h after SAH. As shown in Nissl staining (**A**–**E**), SAH resulted in a significant reduction of surviving neurons, as compared with that in the sham group. After ATX administration, the proportion of surviving neurons was significantly upregulated at 72 h after SAH (**D**). The temporal profile of the neurological scores in experimental groups (**F**). As shown, SAH caused a worse neurological function compared with the sham group both at 24 h and 72 h after surgery. In comparison with the SAH + vehicle group, the ATX treatment group displayed a better neurological function at 24 h, but not durable at 72 h. Values are expressed as the mean ± SEM. ** *p* < 0.01, * *p* < 0.05, ^ns^ *p* > 0.05.

To further see the protection of ATX, the survival neuronal cells at 72 h after SAH were evaluated by Nissl staining. As shown (Figure 7A–D), a large proportion of intact neurons could be seen in the sham group, while numerous damaged neurons with a loss of integrity and dark staining could be seen in the SAH and SAH + vehicle groups. Compared with the SAH + vehicle group, ATX treatment could evidently alleviate the severity of neuronal degeneration (Figure 7E).

The temporal of the neurological function after SAH was also recorded. As shown in Figure 7F, a significant decrease in the neurological scores was observed in the SAH group when compared to the sham group at both 24 h and 72 h after surgery. In comparison with the SAH + vehicle group, treatment with ATX displayed a better neurological function at 24 h, but not significantly at 72 h after SAH. These results suggested that one-time application of ATX might not be sufficient for a durable protection against EBI, and multiple and prolonged use of ATX may be warranted for future SAH brain injury studies.

## 3. Discussion

**Figure 8:**
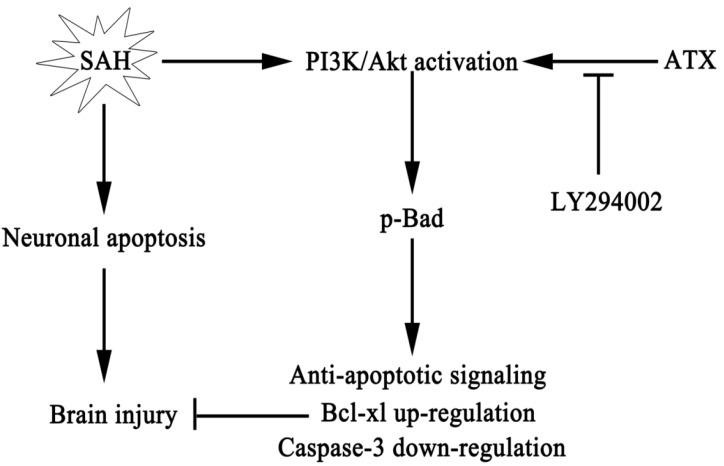
Schematic illustration of ATX and the Akt/Bad pathway mediating anti-apoptotic signals and alleviating early brain injury after subarachnoid hemorrhage (SAH).

To the best of our knowledge, this is the first study to demonstrate a beneficial role for ATX in the brain via a concrete anti-apoptotic pathway. The main findings of this study can be summarized as follows: (1) obvious neuronal apoptosis occurred in this pre-chiasmatic cistern SAH model, and ATX treatment significantly reduced neuronal apoptosis following SAH; (2) the Akt/Bad pathway was activated in the cerebral cortex during the early period of SAH; (3) SAH-induced activation of the Akt/Bad pathway could be further upregulated by administration of ATX, which could inactivate the caspase-3 protein; (4) the PI3K/Akt pathway-specific inhibitor, LY294002, could partially abrogate the anti-apoptotic effect of ATX in EBI via suppressing the activated Akt/Bad pathway; and (5) in addition to the reduced neuronal apoptosis, the neurological dysfunction, brain edema and BBB disruption were also ameliorated after ATX administration (Figure 8). These findings suggested that ATX could provide neuroprotection against apoptosis induced by SAH, at least in part, through the activation of the Akt/Bad pathway.

ATX is a natural compound, widely distributed and occurring in algae and aquatic animals [13]. It has been approved by the U.S. Food and Drug Administration as a feed additive and dietary supplement for a long time [20]. Recently, its multiple properties were widely studied, and increasing studies indicated that ATX has an anti-apoptotic property in different research fields [13,21,22]. In the CNS, Shen *et al.* reported that ATX administration could reduce neuronal apoptosis in a rat ischemic brain injury model [14]. At the same time, in our previous study, we have observed that ATX treatment exerts a neuroprotective effect against SAH via attenuating neural cell apoptosis and the expression of the caspase-3 protein. However, the molecular pathways underlying the ATX-dependent inhibition of the apoptotic pathway were not examined.

The PI3K/Akt pathway is a critical survival mediator in the signal transduction pathways after SAH, and the activation of the PI3K/Akt pathway is a therapeutic target for stroke [23]. Akt is a 57-KDa protein serine-threonine kinase, also referred to as PKB [7]. After phosphorylation, Akt functions through the phosphorylation and inhibition of several substrates, including the pro-apoptotic protein, Bad. Akt phosphorylates Bad on serine-136, which results in its dissociation from Bcl-xL and decreased activation of caspase-3, resulting in cell survival [6,8]. It has been well established that p-Akt expression increases after cerebral ischemia, traumatic brain injury, spinal cord injury and SAH [9,10,11,12]. Moreover, the upregulated p-Akt ameliorated neuronal injury against ischemia, spinal cord injury and SAH [5,9,11]. Consistent with previous studies, the results of the current study demonstrated that the PI3K/Akt pathway was activated in the early period after SAH insults [12,24].

In the previous research regarding the ATX and PI3K/Akt pathway, Kim *et al.* investigated the neuroprotective effects of ATX on H_2_O_2_-mediated apoptotic cell death in cultured mouse neural progenitor cells [17]. They put forward that ATX treatment resulted in the activation of p-Akt and significantly inhibited H_2_O_2_-mediated caspase activation [17]. Similarly, as mentioned by Arunkumar* et al.* in their literature, they investigated the influence of ATX on insulin signaling in obese mice and found that ATX treatment significantly improved p-Akt/Akt ratio [19]. In the current study, administration of ATX resulted in a significant increase in the expression of p-Akt in the cerebral cortex following SAH, which is consistent with previous reports from other disease models [17,19]. Furthermore, ATX treatment promoted the phosphorylation-dependent inactivation of the Bad protein, thereby decreasing the activation of the caspase-3 protein after SAH. In contrast, the PI3K/Akt pathway-specific inhibitor, LY294002, could effectively inhibit ATX-induced expression of p-Akt and p-Bad and partially abolish the anti-apoptotic effect of ATX in EBI. Although, in our study, LY294002 almost completely blocked the anti-apoptotic effect of ATX, the Akt/Bad signaling pathway may be not the only protective pathway. For example, previous studies reported that ATX could inhibit apoptotic cell death via the modulation of the P38 and MEK signaling pathways, the NF-κB pathway, the Nrf-2/ARE pathway, *et al*. *in vitro* and* in vivo* [17,21,25]. In addition to the reduced neural apoptosis, the cerebral edema, BBB permeability and neurological dysfunction, which were the major parts of EBI following SAH, were also ameliorated after ATX administration in our present study. These results support the implication of the Akt/Bad pathway in the neuroprotection of ATX in the early period after SAH.

Cell death, especially apoptosis, is an important component of EBI after SAH [4]. The apoptosis cascades present a number of potential therapeutic opportunities for EBI after SAH. In the current study, we mainly focused on the caspase-dependent apoptosis pathway. Firstly, the caspase-dependent pathway is one of the most important apoptosis cascade to EBI after SAH [2]. Secondly, caspase-3 is the most common mediator in overall apoptosis, including the caspase-dependent and the caspase-independent pathway, and inhibition of caspase-3 activation could reduce cell apoptosis and provide neurovascular protection in EBI after SAH [4,5]. Lastly, caspase-3 is downstream of the PI3K/Akt pathway, and we observed an anti-caspase-3 effect of ATX in EBI after SAH in our earlier study [6,8]. Thus, we chose caspase-3 as the main target of apoptosis in the current study.

However, our study still has several limitations. Firstly, although the neuroprotective effects of ATX and the involvement of the Akt/Bad pathway in EBI have been demonstrated in our study, the potential molecular interaction underlying the initial effects of ATX on the Akt/Bad pathway following SAH is still unknown. Additionally, inhibition of the PI3K/Akt pathway by LY294002 cannot completely reverse the anti-apoptotic effect of ATX in EBI, suggesting that other molecular mechanisms are also involved in the neuroprotective effects of ATX. Secondly, we cannot exclude other properties of ATX implicated in its anti-apoptotic effect in EBI after SAH. For instance, ATX could inhibit glutamate release [14,26], reduce NF-κB translocation to the nucleus [27] and inhibit oxidative damage [28], which may also be involved in the anti-apoptotic function of ATX. Thirdly, in addition to the apoptotic protein, Bad, other pro-survival kinases acting downstream of PI3K, such as serum-and-glucocorticoid-inducible-kinase-1 (SGK1), glycogen synthase kinase-3â (GSK3â) and caspase-9, may also be involved in the anti-apoptotic effects of ATX [29,30,31]. Lastly, in our study, ATX treatment was conducted only once, and we do not know whether multiple and prolonged use of ATX therapy will be effective in the later period of SAH. Therefore, our further studies will be focusing on addressing these issues.

## 4. Experimental Section

### 4.1. Animals

All experimental protocols used for animals (including all surgical procedures) were approved by the Animal Care and Use Committee of Jinling Hospital and conformed to the Guide for the Care and Use of Laboratory Animals published by the National Institutes of Health. Adult male Sprague-Dawley rats (250 and 300 g) were purchased from the Animal Center of Jinling Hospital (Nanjing, China). They were acclimated in a humidified room and maintained on a standard pellet diet with a 12-h light/dark cycle before the experiment. The temperature in both the feeding room and the operation room was maintained at about 25 °C.

### 4.2. Experimental Groups

**Figure 9:**
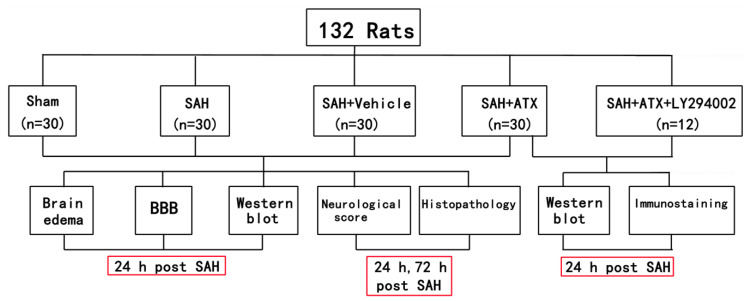
Schematic illustration of the experimental design. SAH, subarachnoid hemorrhage; ATX, astaxanthin.

As shown in Figure 9, animals were randomly allocated into five subgroups: sham group (*n* = 30), SAH group (*n* = 30), SAH + vehicle group (*n* = 30), SAH + ATX group (*n* = 30) and SAH + ATX + LY294002 group (*n* = 12). In the animals of the SAH + ATX group, ATX (20 μL of 0.1 mM dissolved in 10% dimethylsulfoxide) was administered into the left lateral ventricle (0.8 mm posterior, 1.5 mm lateral to the bregma and 3.7 mm below dural) through a 25-μL Hamilton syringe (Shanghai Gaoge Industry & Trade Co. Ltd., Shanghai, China) 30 min after blood injection. Rats of the SAH + vehicle group received equal volumes of vehicle at the corresponding time point. For the rats in the SAH + ATX + LY294002 group, 10 μL of LY294002 solution (50 mM in 10% dimethylsulfoxide) were injected into the left lateral ventricle 30 min before SAH was induced. The dose of ATX was chosen according to our previous study, since we observed beneficial effects on reducing neuronal apoptosis in the SAH model [28].

In the first experimental setting, the animals were killed at 24 h and 72 h after surgery, respectively. Post-assessments included brain edema, blood-brain barrier (BBB) permeability, western blot, neurological function and histopathology study. In the second experiment, the animals were decapitated 24 h after SAH for tissue assays.

### 4.3. Pre-Chiasmatic Cistern SAH Model

**Figure 10:**
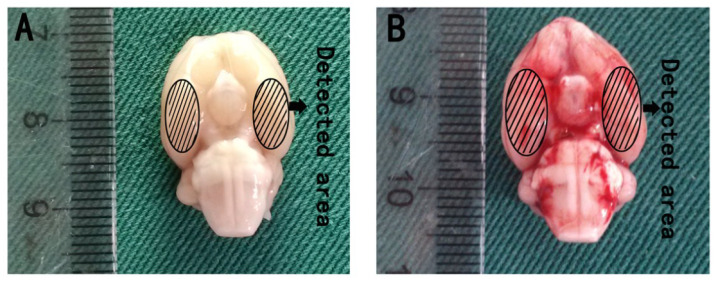
Schematic representation of the cortex sample area for detection. (**A**) Sham group rat brain; (**B**) SAH rat brain harvested 24 h after SAH was induced.

The pre-chiasmatic cistern SAH model was performed as described previously [32]. Briefly, the amount of 0.3 mL non-heparinized fresh autologous arterial blood from the femoral artery was slowly injected into the pre-chiasmatic cistern in 20 s with a syringe pump under aseptic technique. Animals in the sham group were injected with 0.3 mL saline. After operation procedures, the rats were then returned to their cages, and food and water was kept easily accessible. Two milliliters of saline were injected subcutaneously right after the operation. Heart rates and rectal temperature were monitored, and the rectal temperature was kept at 37 °C ± 0.5 °C by using a warm pad when required throughout the experiments. It was observed in the present study that the inferior basal temporal lobe was always stained by blood (Figure 10). Herein, the brain tissue adjacent to the clotted blood was taken for analysis in our study.

### 4.4. Brain Water Content

Brain water content was measured at 24 h after surgery. Rats were anesthetized and decapitated, and the brains were quickly removed and immediately weighed as wet weight. Samples were then placed in an oven for 72 h at 100 °C before determining the dry weight. The brain water content (%) was calculated as a percentage by using the following method: (wet weight − dry weight)/wet weight × 100%.

### 4.5. Blood-Brain Barrier (BBB) Permeability

BBB permeability was assessed by Evans blue (EB) extravasation at 24 h after SAH according to a previous study [32]. Briefly, Evan’s blue dye (2%; 4 mL/kg) was injected over 2 min into the right femoral vein and allowed to circulate for 60 min. Animals were then reanesthetized and perfused transcardially with saline to remove intravascular EB dye. After decapitation, the amount of Evans blue within brain tissue was determined at 620 nm using a spectrophotometer.

### 4.6. Western Blot Analysis

After euthanasia of the rats, the same part of the inferior basal temporal lobe was isolated and processed as described previously [32]. The protein concentration was estimated by the method of Bradford with a standard commercial kit (Bio-Rad Laboratories, Hercules, CA, USA). Equal protein amounts per lane were separated by 10% SDS-PAGE and transferred to a polyvinylidene-difluoride (PVDF) membrane (Bio-Rad Lab, Hercules, CA, USA). The membrane was blocked in 5% skim milk for 2 h at room temperature and incubated overnight at 4 °C with primary antibodies against phosphor-Akt at serine-473 (1:500, R & D, Minneapolis, MN, USA), Akt (1:1000, R & D, Minneapolis, MN, USA), phosphor-Bad at serine-136 (1:500, Cell signaling, Minneapolis, MA, USA), Bad (1:500, Cell signaling, Minneapolis, MA, USA), anti-cleaved caspase-3 (1:1000, Cell signaling, Minneapolis, MA, USA) and GAPDH (1:5000, Bioworld, Minneapolis, MN, USA) in Tris-buffered saline with Tween 20 (TBST) containing 5% skim milk. After, the membrane was washed for 10 min, four times each in TBST, it was incubated with goat anti-rabbit horseradish peroxidase (HRP)-conjugated IgG (diluted 1:1000 in TBST, Bioworld, Minneapolis, MN, USA) for 2 h at room temperature. The blotted protein bands were visualized by enhanced chemiluminescence (ECL) western blot detection reagents (Amersham, Arlington Heights, IL, USA) and were exposed to X-ray film. Developed films were digitized with an Epson Perfection 2480 scanner (Seiko Corp, Nagano, Japan). The quantification of band density was performed using the UN-Scan-It 6.1 software (Silk Scientific Inc., Orem, UT, USA).

### 4.7. Immunohistochemistry

Formalin-fixed, paraffin-embedded sections were subjected to immunohistochemical analysis to determine the immunoreactivity of p-Akt, p-Bad and CC3. For immunohistochemistry, brain sections were incubated overnight at 4 °C with primary antibody against p-Akt (1:100, R & D, Minneapolis, MN, USA), p-Bad (1:500, Cell signaling, Minneapolis, MA, USA) and CC3 (1:100, Cell signaling, Minneapolis, MA, USA) antibody. Sections were then incubated with goat anti-rabbit biotinylated secondary antibody (Santa Cruz Biotechnology, Santa Cruz, CA, USA) and placed in avidin-biotin-peroxidase complex enzyme. Slides were visualized by incubation with 3,3-diaminobenzidine (DAB) and hydrogen peroxide.

### 4.8. Nissl Staining

Tissue sections were stained with Cresyl violet (Nissl), as described [33]. Brain sections were hydrated in 1% toluidine blue for 10 min. After washing with double distilled water, they were dehydrated and mounted with permount. Normal neurons have a relatively big cell body, rich in cytoplasm, with one or two big round nuclei. In contrast, damaged cells show shrunken cell bodies, condensed nuclei, dark cytoplasm and many empty vesicles.

### 4.9. TUNEL Staining

Terminal deoxynucleotidyl transferase-mediated dUTP nick-end labeling (TUNEL) staining was performed according to our previous study [28]. The *in situ* cell death detection kit POD (ISCDD, Boehringer Mannheim, Germany) was used. The procedures were according to the protocol of the kit and our previous study [28].

### 4.10. Cell Counting

From each segment, one slice out of every 6 serial cuttings was selected, and altogether, 6 slices were collected and observed in the light microscope by two independent experienced pathologists blinded to the grouping. The number of positive cells in each section was counted in 10 microscope fields (at ×400 magnification) throughout the identical regions of the studied brain, and the mean per visual field was calculated.

### 4.11. Neurological Scores

Clinical scores were recorded before euthanization based on the independent observations by a veterinarian who was blinded to the experimental groups. Six behavioral activity examinations, including spontaneous activity, symmetrical movements of the four limbs, forepaw outstretching, ability to climb up the wall of a wire cage, body proprioception and vibrissae touch response in the scoring methodology, are used [34]. The total neurological scores ranged from 3 to 18 after adding the scores from all six sectors. Fewer scores indicated worse neurological function.

### 4.12. Statistical Analysis

All data were presented as the mean ± SEM. IBM SPSS Statistics, version 19.0.0, was used for statistical analysis of the data. One-way analysis of variance combined with the Tukey *post hoc* test was used for multiple comparisons. An unpaired *t*-test was used when 2 groups were compared. Statistical significance was inferred at *p* < 0.05.

## 5. Conclusions

In summary, to the best of our knowledge, the present research was the first to demonstrate the effects of ATX on the Akt/Bad pathway in the cerebral cortex after SAH. We found that ATX administration resulted in the magnification of the Akt/Bad pathway and diminution of the degree of secondary brain injury following SAH. The therapeutic benefits of post-SAH ATX administration might be due to its salutary effects on modulating cerebral Akt/Bad survival pathway and anti-apoptotic signaling.
