# Correction: Zhang et al. Astaxanthin Alleviates Early Brain Injury Following Subarachnoid Hemorrhage in Rats: Possible Involvement of Akt/Bad Signaling. *Mar. Drugs* 2014, *8*, 4291

**DOI:** 10.3390/md22120563

**Published:** 2024-12-17

**Authors:** Xiang-Sheng Zhang, Xin Zhang, Qi Wu, Wei Li, Qing-Rong Zhang, Chun-Xi Wang, Xiao-Ming Zhou, Hua Li, Ji-Xin Shi, Meng-Liang Zhou

**Affiliations:** Department of Neurosurgery, Jinling Hospital, School of Medicine, Nanjing University, Nanjing 210000, China; zhangxssp@163.com (X.-S.Z.); njwuqi@gmail.com (Q.W.); lwxzlw@gmail.com (W.L.); njzhangqingrong@aliyun.com (Q.-R.Z.); wangcx02@163.com (C.-X.W.); xiaoming20111386@163.com (X.-M.Z.); shijx52@hotmail.com (J.-X.S.)

**Errors in** **Figures**

The original publication [1] contains errors in two figures.

In Figure 4A, the Nissl image of the SAH + vehicle group was mistakenly selected and used during figure assembling. The other elements of the figure remain unchanged. The corrected Figure 4 is presented below. 

In addition, the Bad blot in Figure 5A was mistakenly sourced from the P-Bad blot. The other elements of the figure remain unchanged. The corrected Figure 5 is presented below.

These changes do not affect the results or conclusions of this study. The authors apologize for any inconvenience caused.

## Figures and Tables

**Figure 4:**
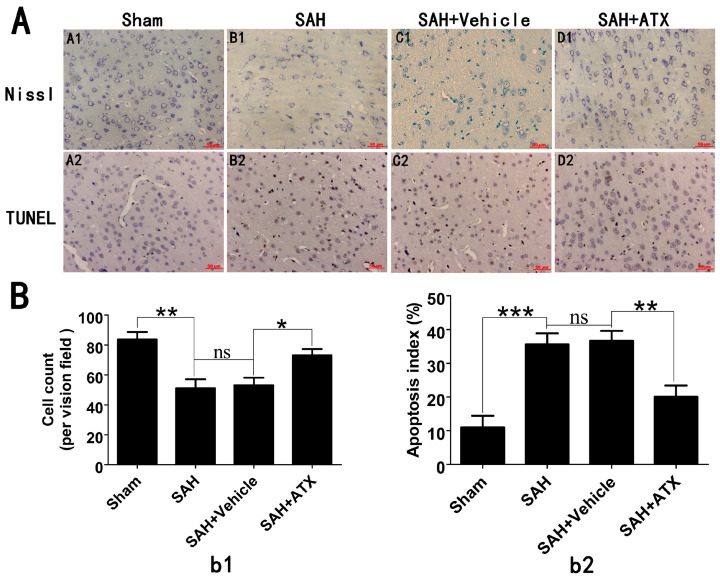
(**A**) Representative photomicrographs of Nissl (**A1**–**D1**) and TUNEL (**A2**–**D2**) staining in the cerebral cortex at 24 h after SAH and (**B**) quantitative analysis of neuronal survival (**b1**) and the apoptotic index (**b2**). As shown in the Nissl staining, in the sham group, the neuronal cell outline was clear and the structure compact, with abundant cytoplasm and Nissl bodies. However, evident neuronal loss and neuronal degeneration were observed in the SAH group and SAH + vehicle groups. Treatment with ATX significantly increased the proportion of surviving neurons. The TUNEL staining showed that the rats in the sham group display rare apoptotic cells in the cortex, while obvious TUNEL-positive cells could be observed in the SAH group and SAH + vehicle groups. In contrast, the proportion of apoptotic cell death decreased significantly in the SAH + ATX group. Values are represented as the mean ± SEM. *** *p* < 0.001, ** *p* < 0.01, * *p* < 0.05, ^ns^ *p* > 0.05.

**Figure 5:**
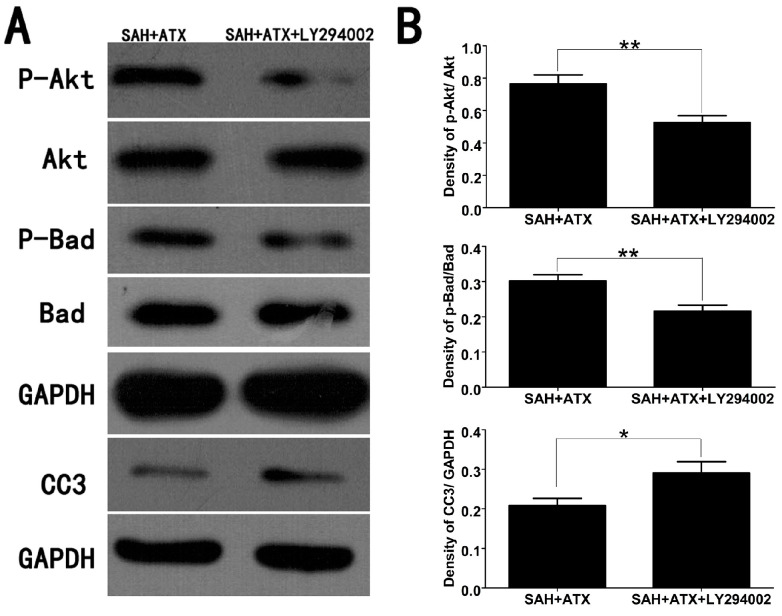
Representative Western blots (**A**) and quantitative analysis of p-Akt, p-Bad and caspase-3 (**B**) in the cortex of the SAH + ATX and SAH + ATX + LY294002 groups. The levels of p-Akt and p-Bad were high in the SAH + ATX group. After LY294002 treatment, the high levels of p-Akt and p-Bad were significantly decreased. In contrast to the low level of caspase-3 in the SAH + ATX group, LY294002 treatment significantly upregulated the level of caspase-3 in the cortex. Results are expressed as the means ± SEM. ** *p* < 0.01, * *p* < 0.05.
